# “Canopy fingerprints” for characterizing three-dimensional point cloud data of soybean canopies

**DOI:** 10.3389/fpls.2023.1141153

**Published:** 2023-03-29

**Authors:** Therin J. Young, Talukder Z. Jubery, Clayton N. Carley, Matthew Carroll, Soumik Sarkar, Asheesh K. Singh, Arti Singh, Baskar Ganapathysubramanian

**Affiliations:** ^1^ Department of Mechanical Engineering, Iowa State University, Ames, IA, United States; ^2^ Translational AI Center, Iowa State University, Ames, IA, United States; ^3^ Department of Agronomy, Iowa State University, Ames, IA, United States

**Keywords:** fingerprints, terrestrial laser scanner, 3D point cloud, phenotypic fingerprint, soybean

## Abstract

Advances in imaging hardware allow high throughput capture of the detailed three-dimensional (3D) structure of plant canopies. The point cloud data is typically post-processed to extract coarse-scale geometric features (like volume, surface area, height, etc.) for downstream analysis. We extend feature extraction from 3D point cloud data to various additional features, which we denote as ‘canopy fingerprints’. This is motivated by the successful application of the fingerprint concept for molecular fingerprints in chemistry applications and acoustic fingerprints in sound engineering applications. We developed an end-to-end pipeline to generate canopy fingerprints of a three-dimensional point cloud of soybean [*Glycine max* (L.) Merr.] canopies grown in hill plots captured by a terrestrial laser scanner (TLS). The pipeline includes noise removal, registration, and plot extraction, followed by the canopy fingerprint generation. The canopy fingerprints are generated by splitting the data into multiple sub-canopy scale components and extracting sub-canopy scale geometric features. The generated canopy fingerprints are interpretable and can assist in identifying patterns in a database of canopies, querying similar canopies, or identifying canopies with a certain shape. The framework can be extended to other modalities (for instance, hyperspectral point clouds) and tuned to find the most informative fingerprint representation for downstream tasks. These canopy fingerprints can aid in the utilization of canopy traits at previously unutilized scales, and therefore have applications in plant breeding and resilient crop production.

## 1 Introduction

Soybean [*Glycine max* (L.) Merr.] canopy characteristics indicate crop growth, development, and health among other characteristics. Canopy traits have traditionally focused on 2-dimensional (2D) features, which is useful in certain instances, for example, canopy coverage (Purcell, 2000), which has frequently been collected with drone high throughput phenotyping (Guo et al., 2021). With the advent of high-throughput crop and plant phenotyping (Araus and Cairns, 2014; Yang et al., 2020; Guo et al., 2021; Jubery et al., 2021; Singh A. K. et al, 2021; Singh D. P. et al., 2021), plant scientists have been able to conduct large scale and time-series investigations on canopy coverage. Additionally, researchers have shown automated or semi-automated extraction of canopy traits; for example, canopy features, including height, shape, color, and texture, can be used for plant stress and disease assessment, estimating total biomass, leaf chlorophyll, and leaf nitrogen (Shiraiwa and Sinclair, 1993; Hunt et al., 2005; Pydipati et al, 2006; Jubery et al., 2016; Bai et al., 2018; Parmley et al., 2019; Parmley et al., 2019). Canopy morphology features, such as shape and size, impact light interception ability, which directly factors into the potential yield equation (Metz et al, 1984; Koester et al., 2014). Canopy characteristics, including height, shape, size, and color, can vary among developmental stages, genotypes, and environments (Virdi et al., 2021). Quantifying the canopy plasticity of a genotype due to changing environmental conditions and variability or similarity among genotypes is valuable for plant breeding applications (Sadras and Slafer, 2012). However, a major hurdle towards effective and full utilization of canopy features is the relatively slow pace of advancement of three-dimensional (3D) canopy features, which provide a “real-world” set of information.

Historically, digital cameras, hyperspectral cameras, and LIDAR have been used to take images and create point clouds of plants (Walter et al., 2019; Herrero-Huerta et al., 2020; Chiozza et al., 2021a; Chang et al., 2022) which are then used to characterize plant traits, leading to a composite plant canopy. Often, methods such as structure from motion or tomographic reconstruction methods are needed to render the 3D point clouds for these traits (Vandenberghe et al, 2018; Storey, 2020). Widely utilized characterization approaches are based on hand-crafted geometric measures, such as plant height, length, breadth, height, area, and volume. Although these geometric features are simple to interpret, they often do not comprehensively represent the spatial variability, for instance, between sample heights and the intricacy of the canopies. Several studies used latent feature representation methods, such as Principal component analysis (PCA) and Neural Network (NN), to characterize the canopy (Gage et al., 2019; Ubbens et al., 2020). Although these features can be used to capture the inherent complexity of the canopies, they are challenging to comprehend since they are difficult to relate to real geometry with low interpretability. There is interest in developing more detailed yet interpretable phenotypic traits for characterizing the crop canopy. Interpretable features are crucial to develop field-testable hypotheses for plant scientists. Most interpretable approaches concentrate on composite characteristics and do not account for individual trait variations. An example approach that offers a middle ground between these two extremes is the elliptical Fourier transformation utilized to describe the complicated geometry of canopy structures (Jubery et al., 2016). However, the use of 3D point clouds can be more exhaustive and informative, motivating researchers to develop holistic phenotyping pipelines (end to end) as well as explore applications of the usage of information from these data. For example, 3D canopy generation has been successfully shown in wheat, *Triticum aestivum* (Paulus et al., 2013; Paulus et al., 2014), rice, *Oryza sativa* (Burgess et al., 2017; Zhu et al., 2018), and other crops (Vandenberghe et al, 2018). These are exciting developments; however, there is still information lacunae on the creation of informative multiscale traits from 3D point cloud data. In this context, non-agricultural disciplines have reported a concept of fingerprinting using point cloud data (Koutsoukas et al., 2014; Spannaus et al., 2021; Wang et al., 2021), but this is lacking in crop production and broader agriculture.

Fingerprinting is a technique for the multiscale characterization of an object by computing a set of unique local characteristics or patterns. Fingerprinting successfully generates unique representations for complex objects in chemistry, geometry, and acoustics (Cano et al., 2005; Capecchi et al, 2020). It was successfully used for the retrieval, recognition, and matching tasks within large molecular and 3D shape databases (Fontaine et al., 2007). Fingerprinting facilitates the representation of a complicated, memory-intensive 3D point cloud as a hierarchically computed, low-dimensional vector. This vector captures both the geometric and topological characteristics of 3D shapes. Computational approaches to computing fingerprinting for 3D objects are broadly based on spectral and non-spectral methods. Spectral approaches utilize the eigenvectors and eigenvalues, referred to as the spectrum, of the Laplace-Beltrami (LB) operator applied to 3D shapes (Reuter et al., 2005). The spectrum is independent of the object’s representation, including the parameterization method and spatial position. Other techniques were developed from LB, for example, Shape-DNA and Global Point Signature (Wu et al, 2022). Probabilistic fingerprinting (Mitra et al., 2006) is a non-spectral fingerprinting technique. It is suitable for determining partial matching between 3D objects. Here, the objects were separated into overlapping patches, unique descriptors were generated for each patch, the descriptors were hashed, and a random subset of the hashed descriptors with a predetermined vector size was chosen as the probabilistic fingerprint. Similar min-hashing techniques (random subset selections) were used to get structural similarity in larger data based on chemistry (Probst, 2018). Hashing aids in the compression of the fingerprinting representation, but this cannot be decoded and is less interpretable. There are application examples of the fingerprinting concept; for example, a phenotypic fingerprint of a soybean canopy was proposed to represent the temporal variation of coarse-scale geometric features, including canopy height and plant length (Zhu et al., 2020b), and it was employed to capture temporal dynamics, identify genotypes with comparable growth signatures, etc. Further, canopy fingerprints enabled large-scale evaluation of the environmental constraints and disturbances that shape the 3D structure of forest canopies (Jucker, 2022). However, thus far, there is no work to define and develop crop canopy fingerprints capturing multi-scale geometric features that could be evaluated and applied in the future in crop modeling, and genomic prediction (Jarquin et al, 2016; Shook et al, 2021a; Shook et al., 2021b), or breeding decisions. Fingerprints are distinctly unique from traditional canopy architecture as they encompass the entire global canopy, while architecture traits are a composite of limited individual traits assessed together.

**Figure 1:**
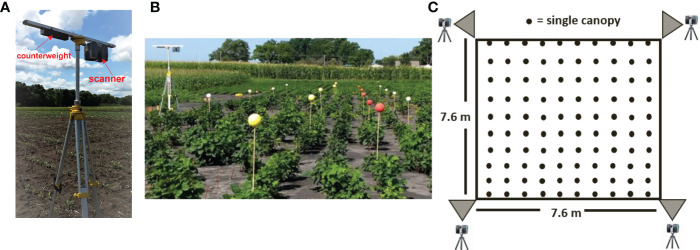
**(A)** Scanning platform: the scanner was mounted on a tripod in an inverted position with an extended bar and counterweight. **(B)** Placement of colored reference markers along the blocks. **(C)** Schematic of the scanning positions, block size, and canopy count per block.

The major contribution of this work is to develop an end-to-end non-spectral interpretable fingerprint generation pipeline for 3D point cloud data of field-grown row crops (**Figure 1**). The pipeline includes point cloud noise removal, registration, plot extraction, and fingerprint generation. We illustrate this approach using a large-scale field experiment through a diversity panel of soybeans. Specifically, we report the construction of canopy fingerprints in soybean using geometric and topological features of the 3D point cloud obtained by a Terrestrial laser scanner (TLS). This is accomplished with an end-to-end pipeline to generate canopy fingerprints of a three-dimensional point cloud of soybean, which is simple to use for feature extraction and utilization in a myriad of applications, including modeling, genomic prediction, ideotype breeding, and cultivar development. For example, the development of unique canopy fingerprints could enable faster and more efficient screening of genetic material for identifying canopy relationships with yield traits (Liu et al, 2016), biotic stress traits such as disease and insects (Pangga et al, 2011), how various canopy levels impact planting density, light interception, and photosynthesis (Feng et al., 2016), enable novel meta-GWAS (Shook et al., 2021a) or improve how crop modeling could predict the ideal canopy fingerprint (Rötter et al., 2015), or fingerprint ideotype, which could then be screened across core collections (Glaszmann et al., 2010) to narrow the pool of experimental genotypes *in silico* prior to *in vivo* evaluation.

## 2 Materials and methods

### 2.1 Laser scanner

The TLS used in this study was Trimble TX5 (Trimble Inc., Sunnyvale, CA, USA) (**Figure 1A**). It is a small and light device (240 mm x 200 mm x 100 mm in size and 5 kg in weight) that can perform measurements at speeds of 1 million points per second. The scanner collects data at a high angular resolution of 0.011 degrees, corresponding to a point spacing of 2 mm at a 10 m scanning range. The scanner emits a 3mm diameter and 905 nm wavelength laser beam and measures the distance between the scanner and the target using the phase-shift principle (Amann et al., 2001). The emitted laser beam is modulated at several frequencies, and the phase shift of all the returned modulations is assessed to increase the accuracy of the distance measurements while storing the intensity of the returned beam. The scanner covers a 360-degree x 300-degree field of view: 360 degrees on the vertical axis is achieved by rotating the scanner head, and a rotating mirror achieves 300 degrees on the horizontal axis. The scanner allows the acquisition of point clouds of 7.1 up to 710.7 million points (MP). The number of points corresponds to the resolution of the measurement. Additionally, the scanner has a built-in camera to capture RGB color values (up to 70 megapixels) and maps them to the corresponding point clouds.

### 2.2 Location, plant materials, and data collection

The experiment was done in a field frequently used for evaluating soybean iron deficiency chlorosis at Iowa State University’s Agricultural Engineering/Agronomy Research Farm, IA, USA, at a latitude of 42.010 and a longitude of -93.735. Four hundred sixty-four soybean cultivars were included in this study. These accessions come from 35 countries and have crop maturities ranging from MG 0 to IV (Mourtzinis and Conley, 2017), along with variable seed weights and stem termination types. In May 2018, the cultivars were hand-planted in hill plots, three seeds per hill with 0.76 m spacing between each hill. Each plot consisted of a single hill replicated three times in the field, with each replication blocked together. No plants were thinned. Preparing a noise-free field is crucial for achieving accurate plant data. To minimize any interference from weeds, we conducted regular weeding at intervals throughout our study. Laser scanning was performed on all plots on the 9th and 10th of August. These scans were conducted 71 and 72 days after planting, and all plants had reached at least the reproductive R3 stage (Fehr et al., 1971) and were entirely opaque from the side, with no leaves visible from the opposite side.

The approximate size of the scanned field was 0.144 hectares (0.355 acres). During scanning, the field was divided into twenty-five 7.6 m x 7.6 m blocks, each containing 100 plots. The scanner was mounted on an 8 kg heavy-duty elevated tripod (Johnson Level, USA) at 2.1 m. For this height, the scanner can see the ground around the base of the farthest canopy within a block. This resulted in the typical scanner to ground distances between 2.1 to 11 m within the block. The device’s scanning resolution was set at 0.5 (angular resolution 0.016 degree), and the expected point distance was 0.6 mm at 2.1 m and 3.1 mm at 11 m from the scanner.

To compensate for the scanner’s field of view restriction of 150 degrees relative to the nadir, or lowest point under the observation lens, the scanner was mounted upside down using a 1.2 m-long bar, as illustrated in **Figure 1A**. This configuration allowed us to scan plots close to the tripod and position the scanner at the edge of each block. Scanning data was captured from four corners for each of the blocks: southwest (SW), southeast (SE), northeast (NE), and northwest (NW). The horizontal rotation limit of the laser scanner was set to 180 degrees, allowing two blocks to be scanned at once.

Before performing scans, Styrofoam spherical targets with a diameter of 0.127 m were placed within each block as reference markers to aid point cloud registration (**Figure 1B**), alignment, and plot identification. The spheres were painted yellow or red and mounted on 1.52 m-tall wooden dowels, which are 0.5 m taller than the expected maximum plant height. The dowels were manually pushed into the soil about 0.15 m deep. Each plot contained six reference markers. A white reference marker was placed at each corner of the block. The position of the reference markers was consistent across all scanned blocks.

Field experiments showed wind speeds to be lowest during the morning hours up until the early afternoon hours. When wind speeds exceeded 14.5 km h^-1^, canopy movement exceeded the uncertainty acceptable for trait measurement. As a result, scanning took place between 9 a.m. and 2 p.m., or when wind speeds were 14.5 km h^-1^ or lower, to ensure the point cloud’s quality was not compromised. While the optimal lighting condition for scanning is at noon, when sunlight is evenly distributed across the scanning area, field experiments demonstrated that overcast lighting also resulted in high-quality point cloud data. We avoided operating the scanner in the early mornings or late afternoons when direct or bright sunlight reflected from plant materials would cause laser signal saturation, resulting in erroneous points synonymous with glare in 2D photography.

Validation data consisting of plant height and canopy area were collected on August 8th. Plant height was recorded as the distance between the soil line at the base of the stem and the topmost leaf. The canopy area was defined as the visible area of the canopy from the nadir described in detail below.

On each plot, plant height was measured manually with a meter stick. The canopy area was measured on a subset of the plots as follows: First, a digital camera (Zenmuse X5 camera with a lens focal length of 45 mm)) mounted on a drone (Matrice 600 Pro) captured RGB images of the plots flown at 30m with 80% overlap and stitched together using Pix 4-D stitching software. We used an in-house Python script to extract individual plots from the stitched orthomosaic image, using the geolocation data obtained from the ground control points (GCP) and the RTK GPS mounted on the UAV. Next, we converted the images from RGB to the HSV color space, and the canopy was separated from the ground by applying a threshold to the Hue (H) color channel. We experimented with different threshold values for the Hue channel and found that the hue value worked best for our case. The canopy area was then calculated by determining the total number of non-zero (canopy) pixels and converting this value to m^2^. To obtain the conversion factor from pixels to m^2^, we measured a predefined marker in the images.

### 2.3 Point cloud processing pipeline

**Figure 2:**
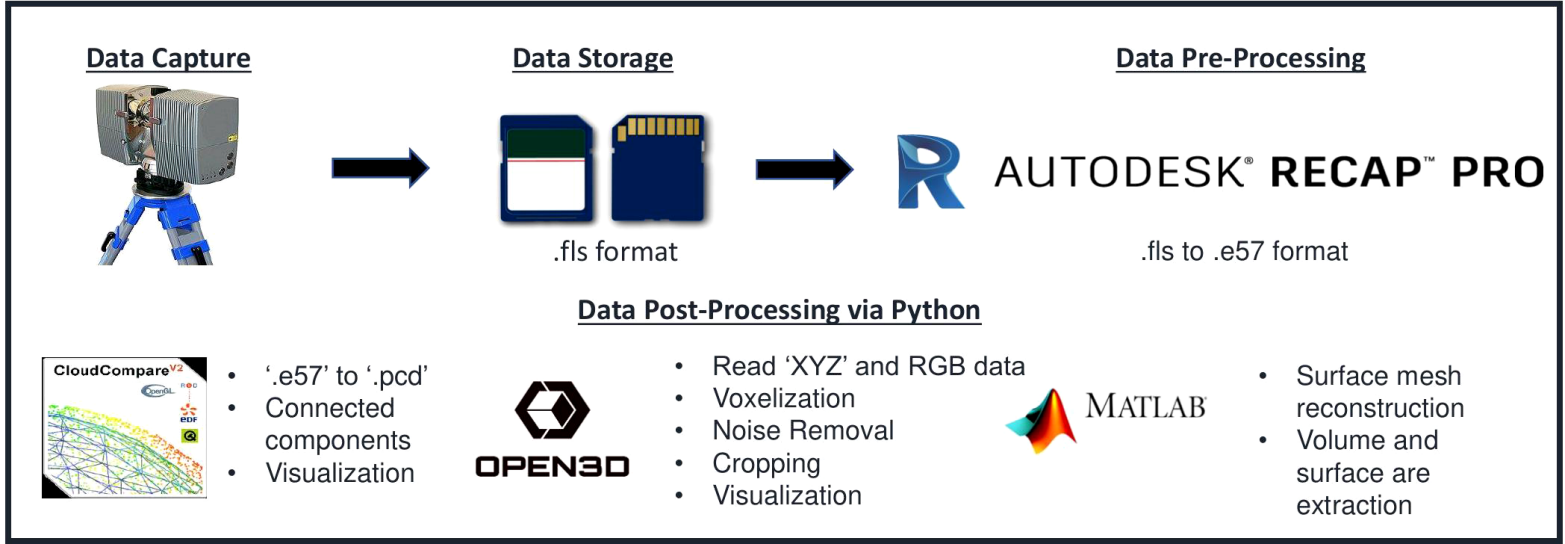
Data Processing Pipeline: Several applications were utilized in the pipeline. Autodesk recap pro was used to convert a scanner-vendor-specific file format to a generic one. CloudCompare and Open3D were employed for noise removal, voxelization, registration, and segmentation.

The pipeline was built using Python 3.7.3 and various other programs and packages, including Autodesk Recap 4.2.2.15, Cloud Compare 2.12.4 (Girardeau-Montaut n.d.), Open3D 0.11.2 (Zhou et al., 2018), and MATLAB 2019a. MATLAB and Cloud Compare were used *via* the command line interface and incorporated into the Python script *via* the Python subprocess library. The specific tasks carried out by these packages are depicted in **Figure 2**. The point cloud data was converted from FLS (Faro) to PCD (Point Cloud Library) format using Autodesk Recap Pro and Cloud Compare. Then, point cloud processing and trait extraction were performed using Open3D, Cloud Compare, and MATLAB. This included cropping, voxelization, registration, noise removal, segmentation, and surface mesh reconstruction.

#### 2.3.1 Point cloud file format conversion

The Trimble TX5 saves point cloud data in the FLS format, which is incompatible with the subsequent point cloud processing software. To ensure compatibility, the point cloud data format was converted from FLS to E57 using the Autodesk Recap Pro software. The E57 file format is a compact, vendor-independent format for storing point clouds, images, and metadata generated by 3D imaging systems such as laser scanners. Additionally, the E57 format retains the RGB component of the point cloud data. Finally, using Cloud Compare, the E57 files were converted to the PCD file format, which includes the Euclidean x, y, and z coordinates of each point and the RGB color value associated with each point.

#### 2.3.2 Region of interest cropping

**Figure 3:**
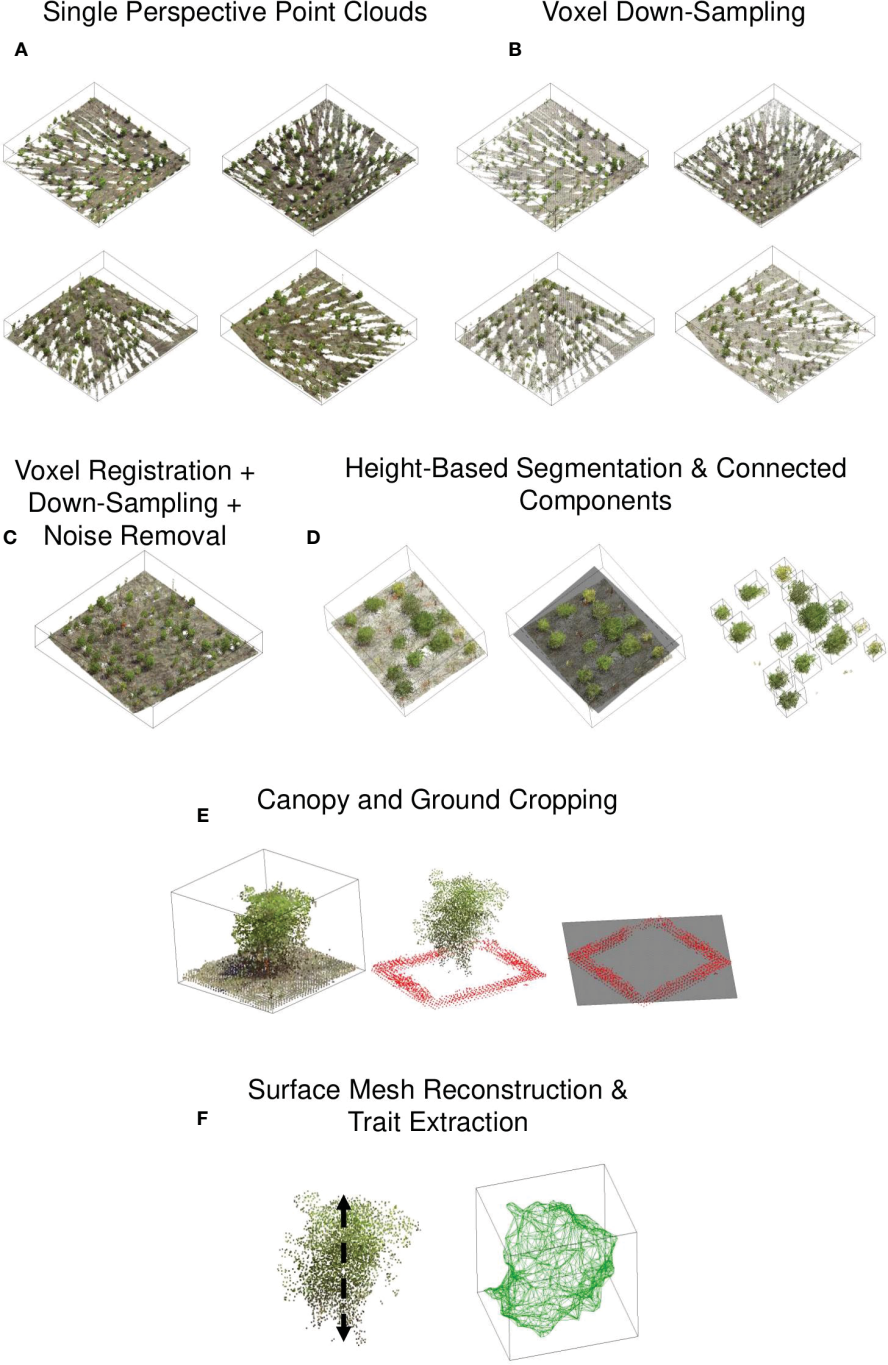
Point cloud processing pipeline: **(A)** The scanner captured the point cloud of a block at four corners of the block. The density of the point cloud is greater in proximity to the scanner. **(B)** Each point cloud was downsampled to reduce disparities in point cloud density. **(C)** The point clouds were registered, and the noise was removed. **(D)** Individual canopy detection was identified using height-based segmentation and connected components algorithm, **(E)** Ground point cloud was removed by identifying visible ground points around the canopy, **(F)** For the canopy point cloud, a triangular surface mesh was generated and the traits, including area, volume were computed.

From the converted point cloud data, the region of interest, a block of the field, from each scan was automatically cropped out using the white-colored reference markers placed at the block’s four corners. The markers were identified by separating points whose normalized R, G, and B color values are close to 1 and have a z coordinate (vertical direction, opposite of the gravity) value greater than 1 m. The z-value constraint was used to eliminate other white objects, such as orange and white plot stake identifiers. Then, the four white markers were identified as distinct objects using the connected components algorithm. Finally, the block was cropped out using the four markers’ mean x and y coordinates (**Figure 3A**).

#### 2.3.3 Homogenization

Due to the variable distances between the scanner and the plots, the point cloud density for a single plot captured from four different corners/perspectives varied (**Figure 3B**). This disparity can cause problems in mesh generation and skeletonization (Labussière et al., 2020; Xia et al., 2020). To reduce the disparity in point density, we voxelized the point clouds. The density of a point cloud is homogenized *via* uniform subsampling or voxel downsampling. We chose the latter method because it is more rigorous in ensuring uniform point distances and is invariant to the distribution of points within the sampling distance. It downsamples a point cloud uniformly using a regular voxel grid of 5 mm resolution. Briefly, voxels are used to group points, and each voxel generates an exact one point by averaging all points within an occupied voxel. Each point contains Euclidean x, y, and z coordinates and R, G, and B values.

#### 2.3.4 Registration

Each block’s four voxelized point clouds were co-registered and merged to form a single point cloud (**Figure 3**). The registration was carried out using the Cloudcompare ‘Align’ tool. We interactively identified a pair of color spheres in the point clouds, and then based on the center of the selected color spheres, the point clouds were aligned by rigid body transformation, ensuring the average root mean squared values of the distances between the paired points after registration is less than 0.01 m. When the preceding procedure failed to produce satisfactory results, we used the iterative closest point (ICP) algorithm to achieve fine registration. The tool can register up to two-point clouds in a single registration. As a result, three registrations were necessary to merge the four perspectives into a single cloud. The final registered point cloud contained duplicate points, and their density was inconsistent. Therefore, it was voxel-downsampled to restore the uniform point density in the registered point cloud.

#### 2.3.5 Noise removal

Due to the so-called edge effect, in which a laser beam is partially intercepted at an object’s edge, and the remaining beam travels further to collide with other objects or passes through the canopy, phase-shift lidar instruments, such as the Trimble TX5, are more prone to generate noisy spurious points *via* range averaging. Additionally, poor co-registration of point clouds and wind-driven movement of the plants can introduce noisy points.

A statistical-outlier-removal algorithm was used to remove noise in the registered point cloud (**Figure 3C**). The algorithm begins by calculating the average distance between each point and its (k) closest neighbors. Then it discards points whose average distance exceeds a predefined threshold, µ+ασ. µ and σ denote the mean and standard deviation of the average distances, respectively, and α is a parameter that can be tuned. The smaller the value of α, the more aggressive the point removal. By monitoring the deviation of a trait value (canopy height) for various combinations, the number of nearest neighbors, k and α, were selected.

#### 2.3.6 Plot segmentation and ground .removal

The visible ground points between the plots were used to segment each plot and remove the ground. A plane (z = f (x, y)) was fitted to the registered point cloud, and the points above the fitted plane were retained (**Figures 3**). The plane passes through the middle of each plot, and the points above the plane comprise the top portion of the canopies. Each canopy top was labeled using the connected component algorithm, and each component’s mean x and y coordinate was considered the plot’s approximate center. The surrounding points within a square band of width 0.1 m and inner length 0.1 m are the faithful ground points for each plot. Finally, a plane was fitted through the ground points, and the points above the plane were considered the canopy.

### 2.4 Trait extraction

Height, volume, and surface area were extracted from the segmented canopy point clouds (**Figure 3**). The canopy height was determined by subtracting the minimum z-value in the canopy points from the mean z-value of the top 3% of canopy points. The choice of using the top 3% was based on a heuristic approach, as it yielded the closest agreement with the ground truth values (See **Supplemental Material (S1)**). To calculate volume and surface area, canopy points were bound into a tight ‘watertight’ triangular mesh using MATLAB’s trisurf algorithm, and the volume and surface area were calculated using Python’s trimesh library. Traits of the projected 2D outline of the point clouds were also extracted. First, the 3d point cloud of the canopy was projected onto the plane of interest. The projected points’ boundary/contour was considered the 2D canopy outline. Area, aspect ratio, roundness, circularity, and solidity of the outline are defined as ( Jubery et al., 2016):

• aspect ratio = major axis of the best-fit ellipse on the outline: minor axis of the best-fit ellipse on the outline; the ratio of the major to the minor axis of the best-fitted ellipse on the outline;
• roundness = 4 ∗ Area/(pi ∗ MajorAxis^2^); it indicates the closeness of the shape of the outline to a circle;
• circularity = 4 ∗ pi ∗ Area/(Perimeter)^2;^ it indicates the closeness of the form of the outline to a circle;
• solidity = Area/Convex Area; it is a measure of the compactness of the object.

### 2.5 Canopy fingerprints

**Figure 4:**
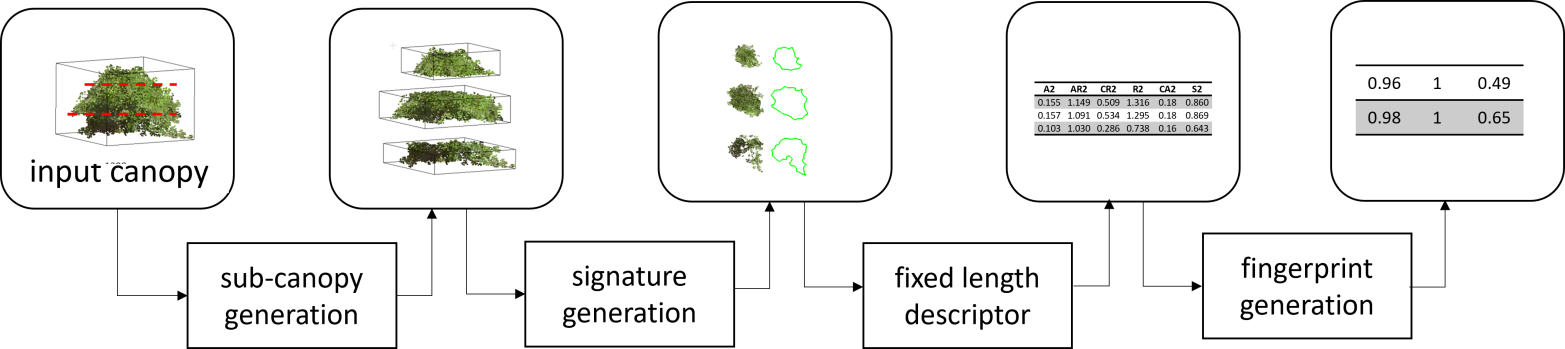
Overview of soybean canopy fingerprinting. Each canopy was subdivided into a predetermined number of sub-canopies, and the signature of each sub-canopy was extracted using several features, which were then arranged in a vector format and normalized with respect to the center sub-canopy features to represent the fingerprint of the canopy.

Fingerprints are a way of representing complex physical objects mathematically. It can be used to illustrate various features on a local scale in a hierarchical and/or multi-scale way. Mathematical representation enables statistical or machine learning techniques to determine the similarity, signatures, and relationships between groups of objects. Here, we fingerprint a canopy by encoding it as a collection of sub-canopy-level features. For example, to fingerprint the shape of a canopy, we divide it into 2n+1 equally divided sections (sub-canopy). Here, we divide the canopy in the height direction into the 2n+1 sections. We then generate the signature of each sub-canopy using several geometric traits and normalize the traits concerning the traits of the center (n^th^) sub-canopy. Finally, we represent normalized traits in a vector format to generate the fingerprint. Here ‘n’ is a tunable parameter that depends on the complexity of the canopies and intended downstream tasks involving the fingerprints (**Figure 4**).

## 3 Results and discussion

### 3.1 Parameters/conditions for TLS scanning and point cloud processing

Results showed that the point count (an indirect estimate of point density) of a canopy varies by its distance from the scanner, with as much as a 50% reduction when a canopy is close to the scanner versus when it is at the farthest possible distance from the scanner. However, this study circumvents the point density effect by registering multiple perspectives of the same scanned area. Thus, if the point density of a canopy near the scanner decreases as the scanner is moved farther away, the points lost can be recovered by scanning from a distinct perspective closer to the canopy (See **Supplemental Material (S2)**).

We used statistical outlier removal to reduce noise from the point cloud. Outlier selection is dependent on the values of two parameters: k, the number of neighbors, and alpha, the standard deviation ratio. We investigated the effect of 40 different combinations of these parameters on the extracted canopy height, including five values of the number of neighbors (k = 8, 16, 24, 32, 40) and eight levels of the standard deviation ratio.

**Figure 5:**
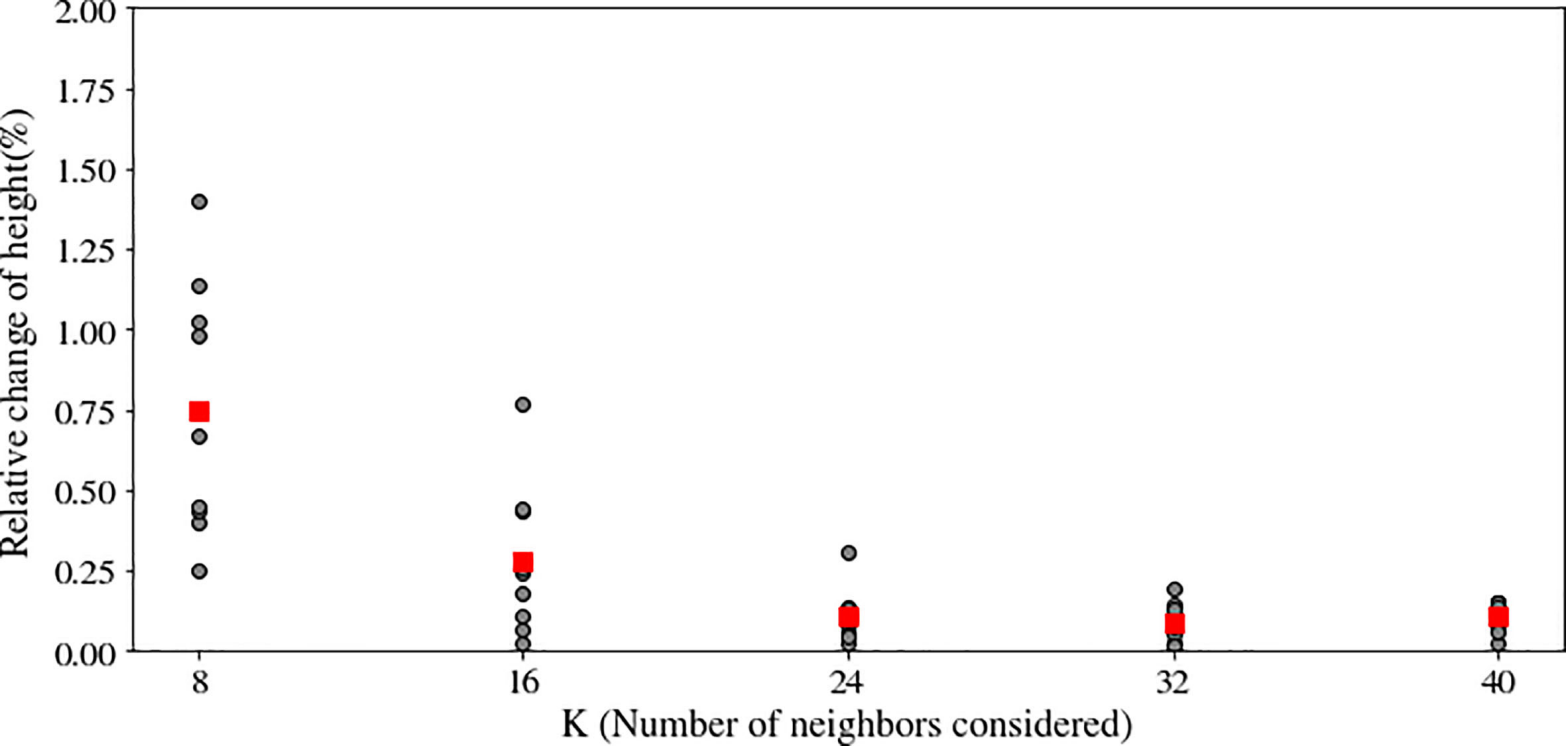
Effect of the number of neighbors (K) in the noise removal algorithm on the plant’s height after noise removal. A large neighborhood size may eliminate both the actual canopy point cloud and noise, but a small neighborhood size may result in the preservation of noisy points. Optimal K was determined when increasing K had a negligible effect on plant height.

The change in nearest neighbor parameter, k, from 8 to 16, average canopy height difference changed significantly after increasing (**Figure 5**). However, there were no significant changes in the average height difference for the remaining experiments (k = 32, k = 40). Additionally, following each experiment, visual analysis of the canopy point cloud revealed that most outliers were removed at k = 24. When k ≥ 24 and the standard deviation ratio was 0.075, no visually discernible changes in canopy structure occurred. Thus, the number of nearest neighbors, k, and the standard deviation ratio noise removal parameters were set at 24 and 0.075 for all noise removal tasks, respectively.

**Figure 6:**
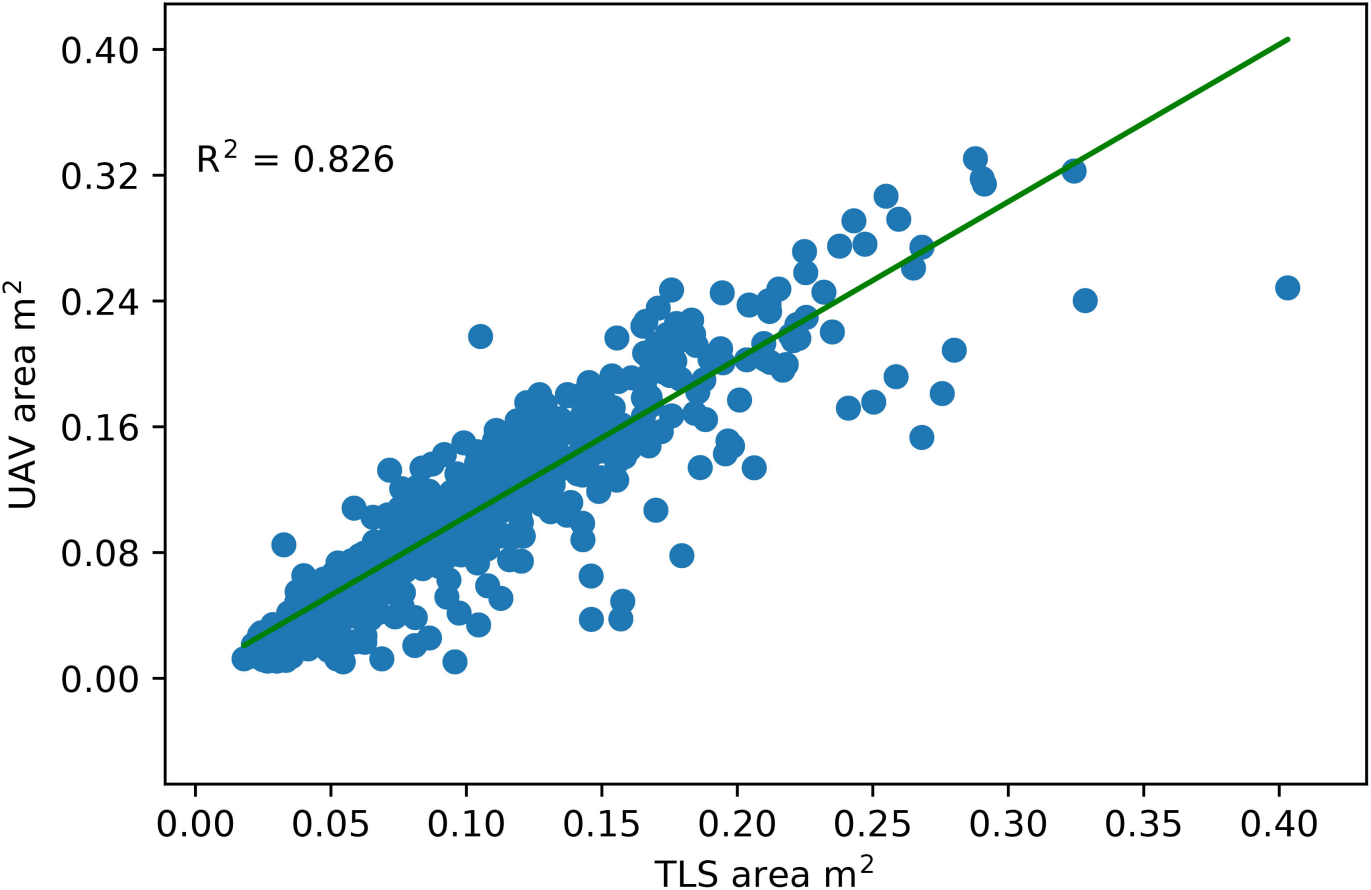
Comparison of the canopy area calculated from images captured by the UAV mount camera with the point clouds captured by the TLS. The TLS point cloud was projected onto a 2D XY plane (Top view), and the area of the closed contour around the projected point cloud was considered a canopy area.

To evaluate the performance of co-registration, we determined the canopy top-view 2D projected area of a subsample of plants and compared it to the ground truth 2D projected area extracted from RGB images. By flattening the z values, the 3D point cloud of the canopy was projected onto the XY plane. A closed polyline represented the boundary/contour of the projected points, and its area was taken as the canopy area. An excellent agreement between the top-view canopy area and the ground truth area with R^2^ = 0.826 (**Figure 6**).

### 3.2 Validation of the extracted canopy height

**Figure 7:**
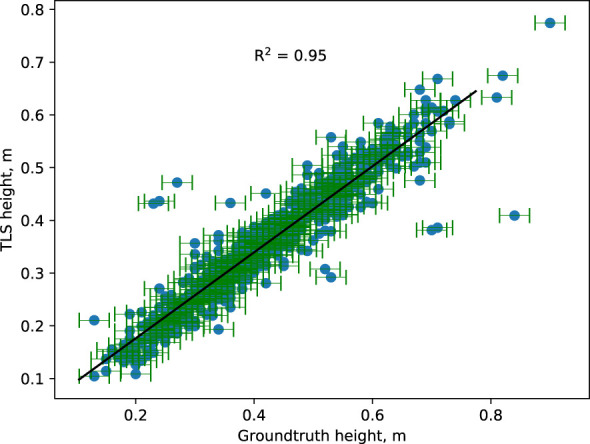
Comparison between manually measured canopy height (ground truth) and automatically measured canopy height from TLS-captured point cloud. The horizontal bar represents the measurement uncertainty associated with ground-truth data. The TLS-based plant height corresponds well with the actual plant height. Extreme outliers are believed to be the result of human mistakes.

The results indicate that the extracted canopy height from the point cloud correlates with manual ground truth measurements taken on the same scanning day (**Figure 7**). Around 95% of the variation observed in extracted height values could be explained by a fitted linear least-squares regression model. Ground truth outliers in canopy height were defined as individuals with a Z-score greater than 2.5 compared to all samples’ mean and standard deviation. Fewer than 2% of ground truth height measurements were considered outliers and were likely human errors in the collection. The deviation of heights from the manual measurements within the outliers ranged from 0.11 m to 0.43 m, with TLS measurements more frequently smaller than ground truth. After visualizing the outlier canopies’ point cloud data, one explanation for the lower TLS height measurements is that occlusions between the measured canopy and neighboring canopies were not detected during data processing. However, manual height measurements of the canopies confirmed that the canopy segmentation and TLS height measurements were accurate (See **Supplemental Material (S3)**). Due to the low outlier rate (less than 2%) and high correlation (95%) between TLS and ground truth height data, TLS-based height extraction is a more robust method for canopy height measurement. This is useful because the TLS based method is automated.

### 3.3 Fingerprinting and implications

**Figure 8:**
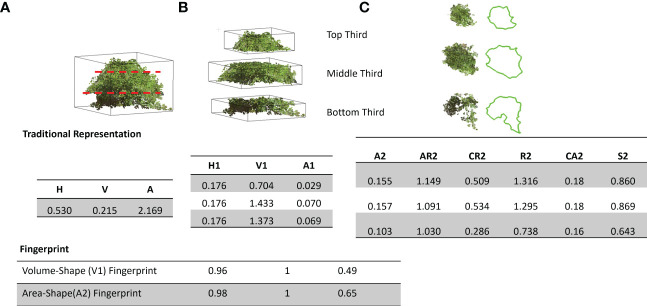
Traditional representation vs. canopy fingerprinting: Top panel: **(A)** Full canopy representation using height (H), volume (V), and surface area **(A, B)** Sub-canopy features including height (H1), volume(V1), Area(A1), **(C)** Sub-canopy 2D features including Area (A2), Aspect ratio (AR2), Circularity (CR2), Roundness (R2), Convex Area (CA2) and Solidity (S2), bottom panel: Fingerprint of the canopy shape Volume and Projected 2D Area (A2).

Typical drone and LiDAR 3D point clouds are often limited to a top-down view of the plant canopy due to collection limitations. **Figure 8** depicts the canopy’s conventional representation, as often shown from the whole plant perspective, and then shows a fingerprint perspective. The fingerprint representation was created by dividing the canopy into three and nine sub-canopies (2n+1, where n = 1 and 4). With these canopy fingerprints, we can find similar-looking canopies for a given canopy or a desired/given shape. Splitting these canopies into sub-canopies enables new opportunities for phenomics and further genomic assessment of cultivars. Traditionally, only the labor and time-extensive method of plant component partitioning would come close to this capability, but still lacked the ability for fingerprinting (Hintz and Albrecht, 1994; Raza et al., 2021). Sub-canopies paired with their fingerprints have the potential to further explore the unique relationships between certain fingerprint types or clusters with known canopy traits such as branching, leaf size, or leaf angle and their relationships with yield and yield component traits (Feng et al., 2018; Bianchi et al., 2020; Moro Rosso et al, 2021).

**Figure 9:**
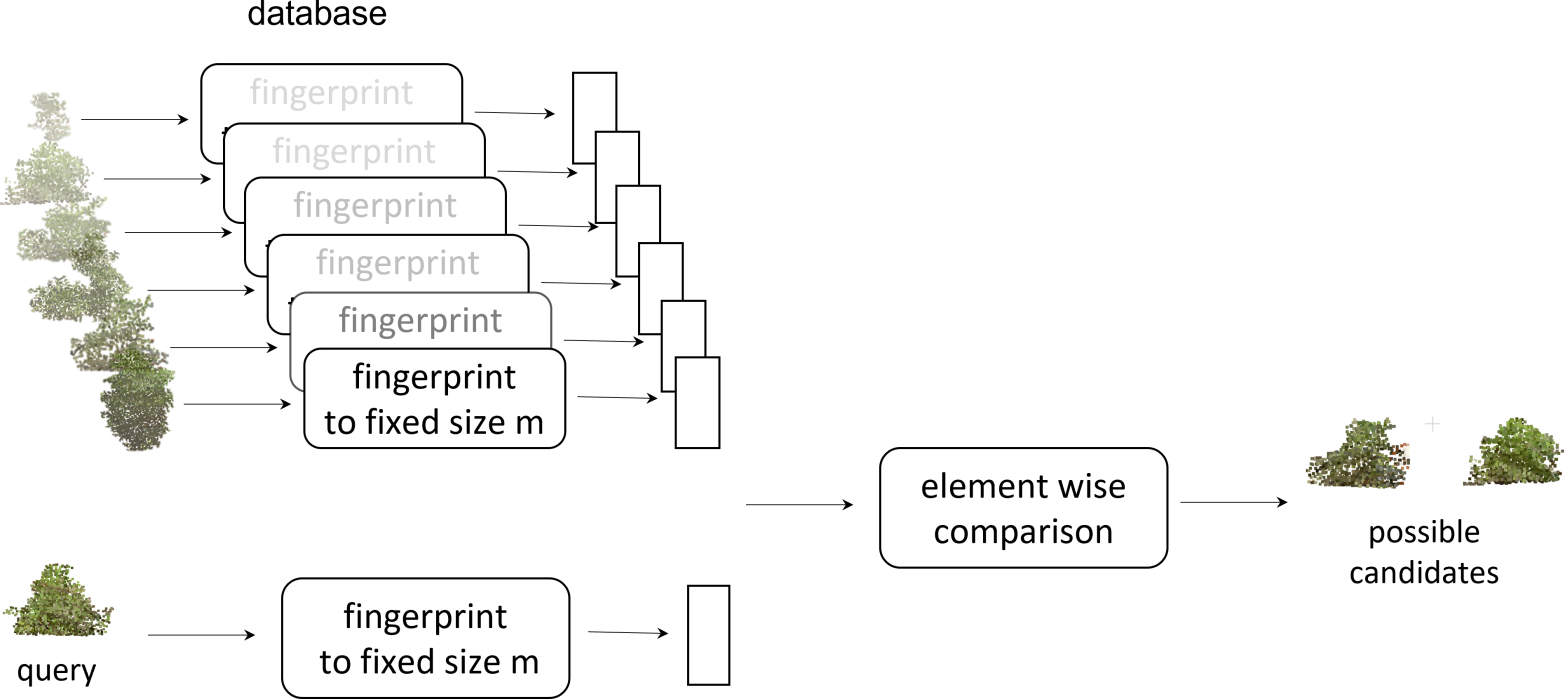
Query of a given canopy: the canopy point clouds database was converted to a fingerprint database. To query a given canopy, a fingerprint of the canopy was generated and then compared with the existing database of fingerprints to identify the possible match.

**Figure 10:**
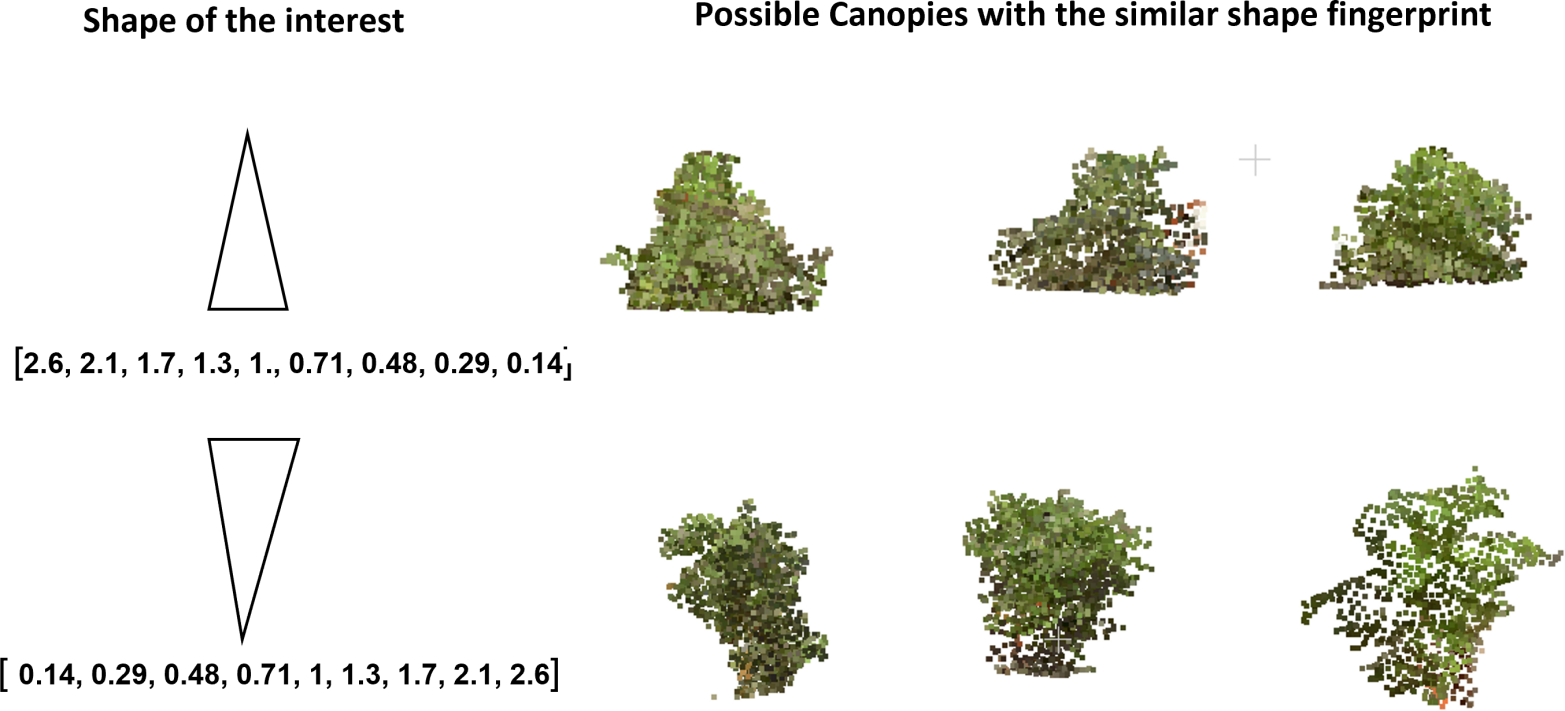
Canopies of given shapes (conical and inverted conical) were queried from the fingerprint database. The candidates look representative of the given shapes.

With digital canopy fingerprints, we can now query a given canopy (**Figure 9**). The canopy point clouds database was converted to a searchable fingerprint database. To query a given canopy, a fingerprint of the canopy was generated and then compared with the existing database of fingerprints to identify the possible match. This capability could enable further in-depth development and exploration of the germplasm resources for ideotypes (Kokubun, 1988; Evangelista et al., 2021; Lukas et al., 2022; Roth et al., 2022). While this work evaluated a single time point, a more in-depth and temporal fingerprint could be developed to evaluate the canopy growth and development across time. These temporal fingerprints could open new insights into agronomic traits (Pfeiffer and Pilcher, 1987; Shiraiwa and Sinclair, 1993), diseases, and pesticide applications (Hanna et al., 2008; Sikora et al., 2014; Nagasubramanian et al., 2019; Viggers et al, 2022), or even develop fingerprint responses to abiotic stress and amendments (Frederick et al., 1991; Anda et al., 2021; Shivani Chiranjeevi et al, 2021; Bonds et al., 2022). An example of searching for potential ideotypes is shown in **Figure 10**, where some canopies with monotonically increasing mass at the top or the bottom are identified using the fingerprint representation. To find a canopy of the desired shape, we constructed a fingerprint of the desired shape and then determined which canopy fingerprints have the least Euclidean distance to the desired fingerprint. This further enables the exploration of canopy fingerprints *in silico* not only in relation to proposed ideotypes but also as a complement to crop modeling. One of the core components of crop modeling is modeling the effect of light interception and radiation use efficiency of the canopy (Edwards et al, 2005; Singer et al., 2011; Zhu et al., 2020a). With canopy fingerprints integrated into a crop model, the theoretical evaluation of more genotypes in the models would be enabled, and stronger models could be developed and could also be expanded to explore environmental impacts and impacts on canopy fingerprints (Chiozza et al., 2021b; Krause et al., 2022).

**Figure 11:**
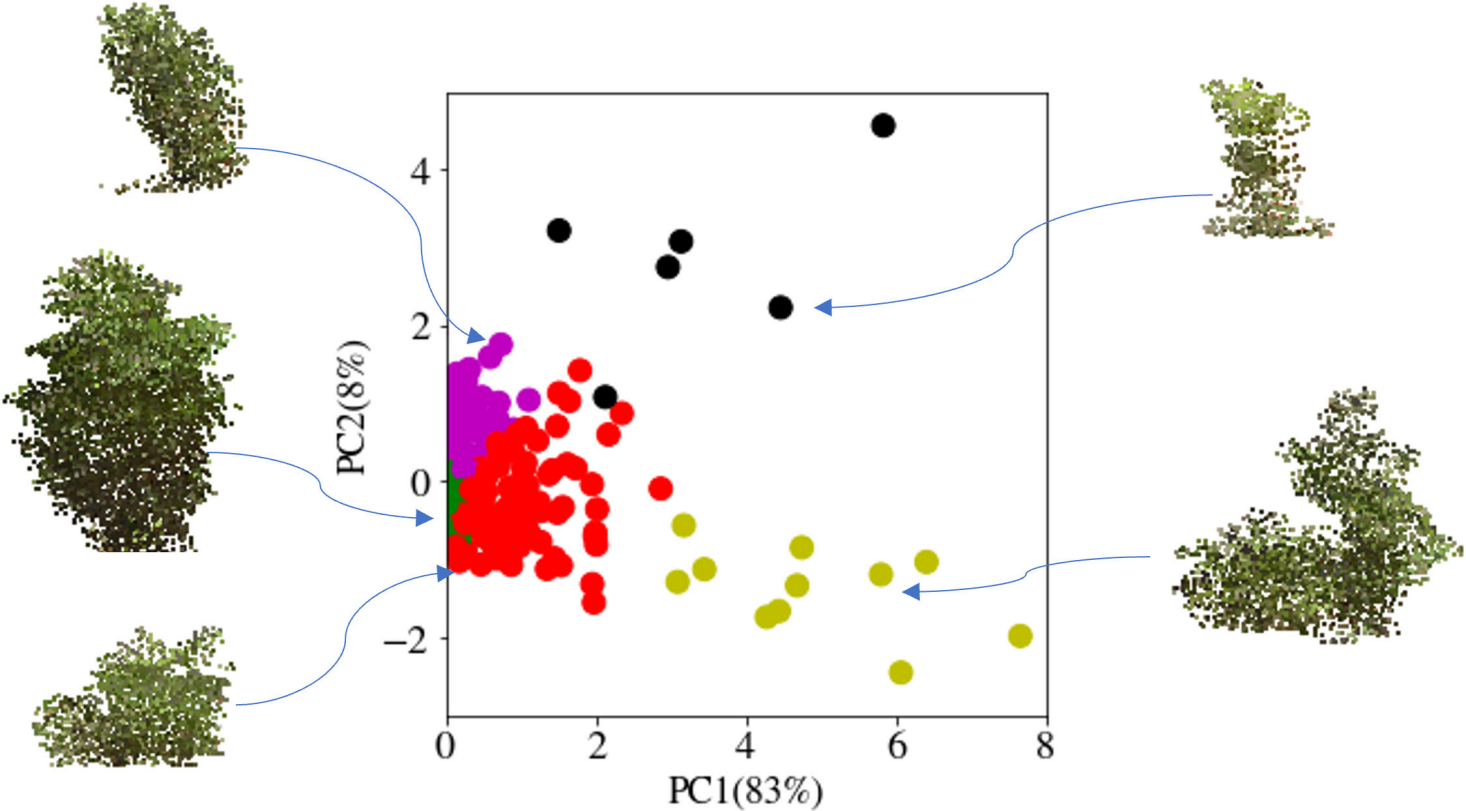
Identifying diversity in the canopy database: The fingerprints of the canopies were clustered and then visualized after dimensionality reduction using principal component analysis (PCA). The cluster-representative samples demonstrate the diversity of the canopies of each cluster.

An aspect of the fingerprinting to be further developed would be including the RGB data in the fingerprints. As apparent canopy color is related to soybean photosynthetic activity yield and plant health (Harrison et al, 1981; Rogovska et al, 2007; Naik et al., 2017; Yuan et al., 2019; Kaler et al., 2020; Rairdin et al., 2022). While the RGB data is already included within the voxels, additional work to evaluate the impact on fingerprint clustering due to color changes within each sublayer will be useful. Evaluating the color differences within each layer could provide an added trait assessment for radiation use efficiency relative to the amount of chlorophyll active in each canopy layer. **Figure 11** shows PCA performed on the fingerprints and clustering. Each cluster’s representative sample looks quite different and shows that the fingerprint can be used to pick diverse samples with the shape analysis alone. However, the inclusion of color data and additional layers, such as horizontal sub-layering, could further enable more detailed fingerprints for assessment and query while still reducing the computation load required for searching canopy fingerprint databases. These methods can parse out canopy features (through their fingerprinting) for a more informative representation of the canopy and the role of various organs throughout the canopy on desired traits, e.g., seed yield. This enables the discovery of new relationships between canopy and organ level features and their impact on yield and yield component traits.

While this work is focused on fingerprints assessed from TLS laser point clouds, the concept of canopy fingerprints could also be implemented with any technology capable of building a full canopy 3D point cloud, such as structured light, space-carving, or full canopy structure from motion (Nguyen et al., 2015; Zhou et al., 2019; Das Choudhury et al., 2020). While we focus on canopy fingerprints, further work should be done to evaluate whole plant fingerprints, especially root system architecture (RSA) fingerprints. While 2D imaging is routine for RSA traits (Falk et al, 2020b; Jubery et al., 2021), there is tremendous interest in 3D imaging of root traits. RSA Fingerprints would further enable a whole plant analysis and efficient query system, and technology such as Xray-CT already enables dense 3D point clouds to be built of RSA (Gerth et al., 2021; Teramoto et al, 2021). Whole plant fingerprints could help meet the need for efficient RSA and canopy modeling, clustering, and assessment (Falk et al., 2020a; Carley et al., 2022a) while further exploring the root and shoot relationships to critical traits such as nodulation (Carley et al., 2022b). Irrespective of shoot or root fingerprints, there is tremendous potential for using this information to ID specific accessions and characterize germplasm collection (Azevedo Peixoto et al., 2017), cluster them based on their canopy features, develop relationships between agronomic, disease, or stress-induced traits, and modularize canopy features for their integration in trait development.

## 4 Conclusions

This study proposed an end-to-end fingerprint generation pipeline from a 3D point cloud of diverse soybean canopies grown on hill plots. The pipeline includes point cloud noise removal, registration, plot extraction, and fingerprint generation. Canopy fingerprinting is a generic and powerful approach to constructing interpretable, multi-scale, and/or hierarchical geometric traits from 3D point cloud data. This approach is a useful middle ground between conventional approaches of extracting coarse scale (i.e., full canopy scale) geometric features that may not comprehensively capture the spatial distribution of the canopy and the more recent approaches of directly compressing the point cloud data that produce difficult to interpret features. The generated fingerprints were used to query canopies of specific shapes to the group and identify similar canopies, which could be useful for future work in further identifying the relationships between canopy, agronomic traits, and yield relative to proposed ideotypes in varying climate scenarios. Canopy or whole plant fingerprinting could be used as a pre-classifier for a complete shape-based retrieval system. It could be used as a pseudo-leveler for self-supervised model training (REF) or useful in situations of limited annotation to train ML models (Kar et al., 2021; Nagasubramanian et al., 2021). Fingerprints could be added as a semantic tag (metadata) to the point cloud and can be queried instead of opening the data, and can also be used for privacy-preserving deep models if data sharing is challenging (Cho et al., 2022). Fingerprinting also serves as a promising tool to store and quantify the inter-genotype or inter-environment variability. In combination with crop models and further development of voxel RGB data, these fingerprints could enable vast and rapid assessment of *in silico* genotypes for future experimentation in addition to the already improved searchability that fingerprint databases provide. As the fingerprint is based on simple sub-canopy level features, it has some limitations, and the proposed framework is sensitive to rigid transformations. If an upright plant becomes tilted, we get different fingerprint representations. However, this could be useful for estimating agronomic traits like lodging. Additionally, the vector of features as a function of plant height could be used for functional GWAS to explore putative loci with multi-scale canopy features. While our pipeline is built on TLS, future applications need to explore drone- and ground-based phenotyping (Gao et al., 2018; Guo et al., 2021; Riera et al., 2021). Plant phenotypic fingerprints serve as a novel opportunity to offer a diverse advantage to the future of high throughput phenotyping serving as a useful tool for data curation, cultivar selection, evaluation, and additional experimentation. Integration of canopy fingerprints with machine learning models can further advance the field of phenomics and cyber-agricultural systems (Singh et al., 2016; Singh et al., 2018; Singh A. K. et al., 2021).

## Data availability statement

The raw data supporting the conclusions of this article will be made available by the authors, without undue reservation.

## Author contributions

TY and TJ: Conceptualization; Data Collection; Data curation; Formal analysis; Investigation; Methodology; Software; Validation; Visualization; Writing-original draft; Writing-review and editing. CC: Writing-original draft; Writing-review and editing. MC: Field experimentation; Data Collection; Writing-review and editing. SS: Funding acquisition; Resources; Supervision; Writing-review and editing. AKS: Funding acquisition; Resources; Experimentation; Writing-original draft; Writing-review and editing. AS: Conceptualization; Funding acquisition; Resources; Methodology; Supervision; Writing-review and editing. BG: Conceptualization; Funding acquisition; Investigation; Methodology; Project administration; Resources; Supervision; Writing-review and editing. All authors contributed to the article and approved the submitted version.

## Supplementary material

Supplementary Figure 1 R-squared (R²) values of the predicted TLS height against the ground truth values, with respect to varying % of top canopy points. The results showed that the R² value was highest when canopy height was calculated using the top 3% of the canopy points.

Supplementary Figure 2 Variation of point count of a canopy relative to its distance from the scanner.

Supplementary Figure 3 Examples of height measurement differences between TLS and Manual. The images depict interactive TLS (3D point cloud)-based height measurement within the CloudCompare software.

Supplementary Figure 4 This histogram displays the distribution of canopies based on the number of points they contain. The data pertains to 464 soybean cultivars including 450 plant introduction (PI) lines that were studied, representing a diverse range of maturities, seed weights, and stem terminations, and originating from 35 different countries. Data was obtained from one or two replicates per cultivar.
