# Supplementary material for: “Canopy fingerprints” for characterizing three-dimensional point cloud data of soybean canopies
Source: Front Plant Sci. 2023 Mar 29;14:1141153. doi: 10.3389/fpls.2023.1141153 (PMC10090282; doi:10.3389/fpls.2023.1141153)
Supplement: Supplementary Figure 1 — R-squared (R²) values of the predicted TLS height against the ground truth values, with respect to varying % of top canopy points. The results showed that the R² value was highest when canopy height was calculated using the top 3% of the canopy points. [file DataSheet_1.docx]

**‘Canopy fingerprints’ for characterizing three-dimensional point cloud data of soybean canopies**

Therin J Young^1#^, Talukder Z Jubery^2#^, Clayton N Carley^3^, Matthew Carroll^3^, Soumik Sarkar^1,2^, Asheesh K Singh^3^, Arti Singh^3*^, Baskar Ganapathysubramanian^1,2*^

^1^Department of Mechanical Engineering, Iowa State University, Ames, IA, USA

^2^Translational AI Center, Iowa State University, Ames, IA, USA

^3^Department of Agronomy, Iowa State University, Ames, IA, USA

# Contributed equally

*** Correspondence:**Baskar Ganapathysubramanian: [baskarg@iastate.edu](mailto:baskarg@iastate.edu);

Arti Singh: [arti@iastate.edu](mailto:arti@iastate.edu)

**Supplementary Figures**


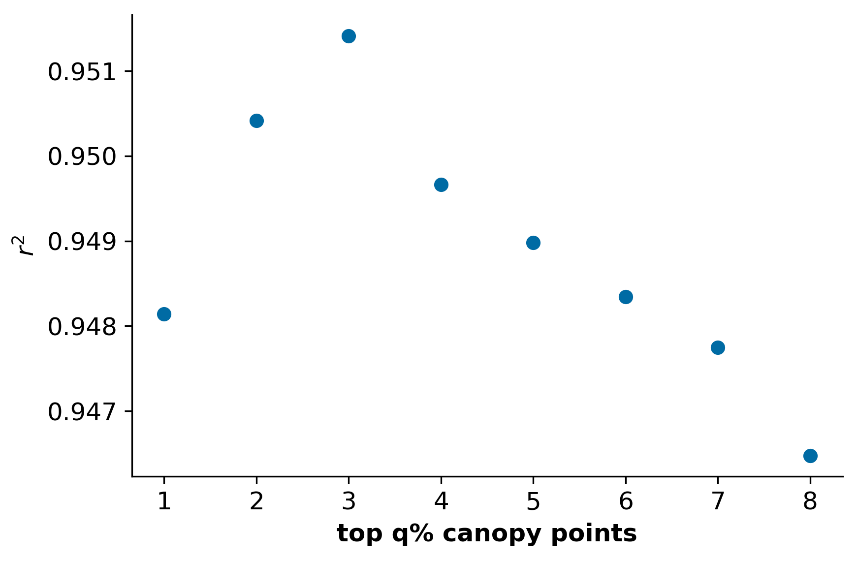


S1: R-squared (R²) values of the predicted TLS height against the ground truth values, with respect to varying % of top canopy points. The results showed that the R² value was highest when canopy height was calculated using the top 3% of the canopy points.

**
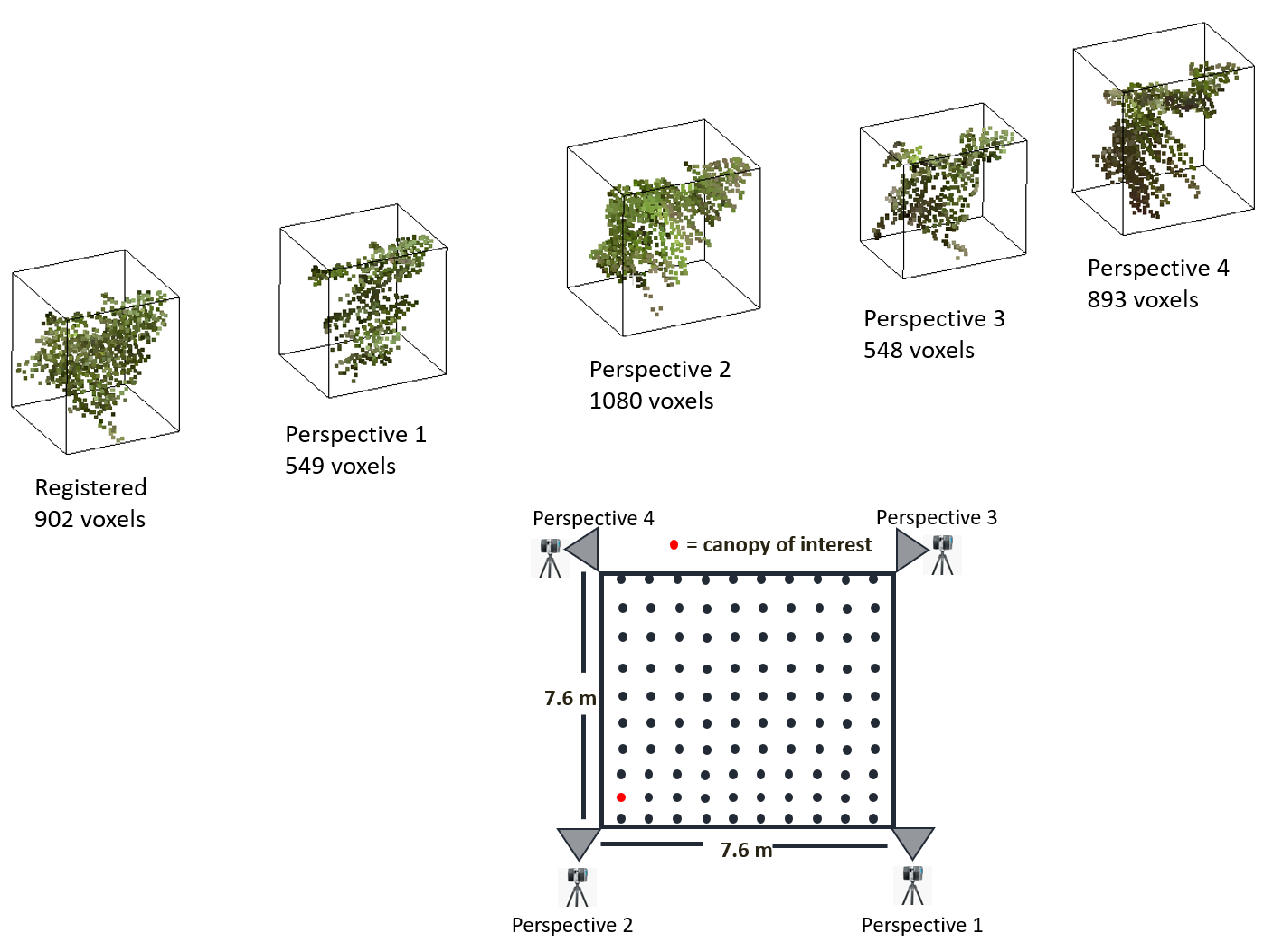
**

**S2:** Variation of point count of a canopy relative to its distance from the scanner.


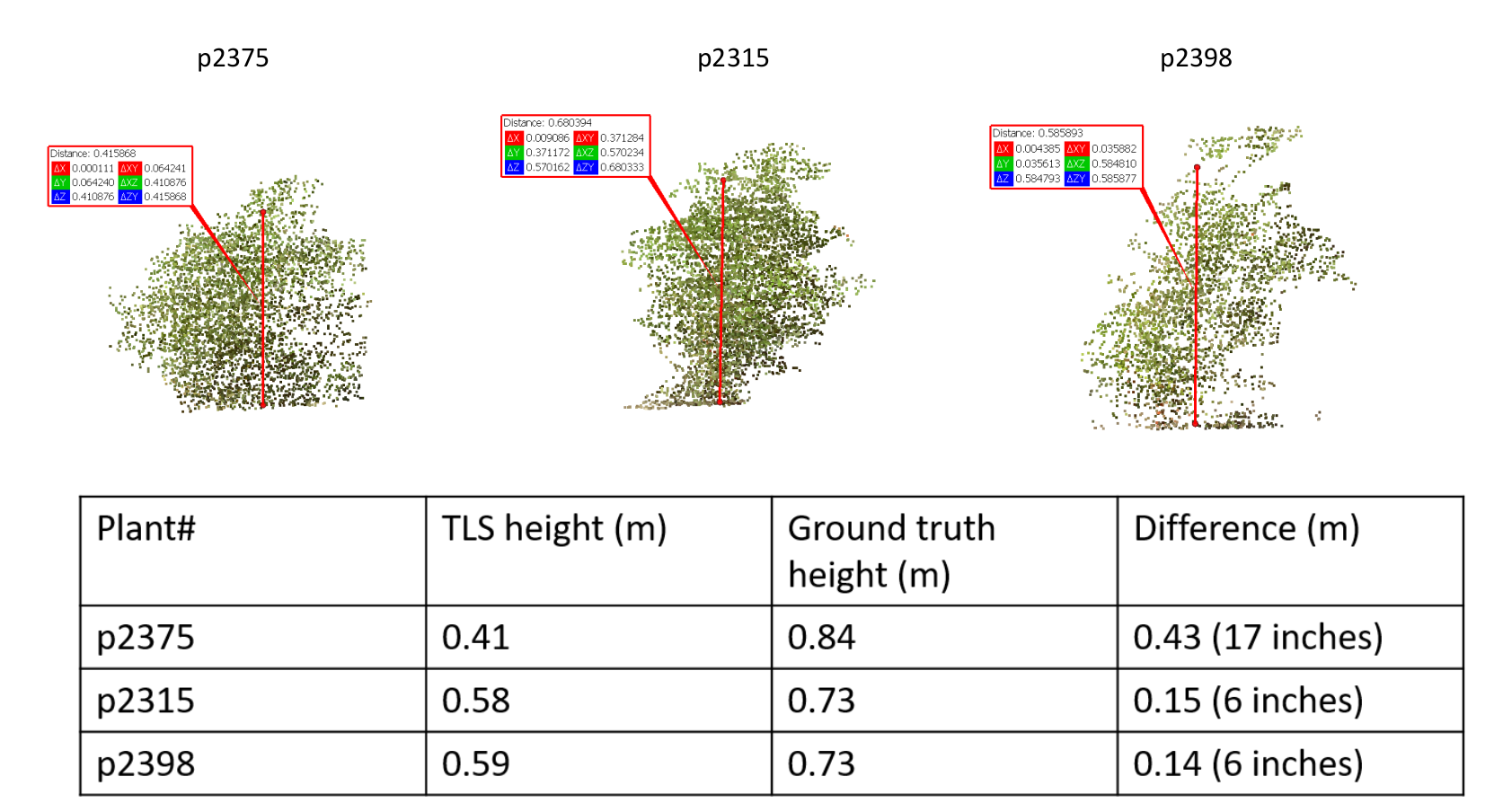


**S3:** Examples of height measurement differences between TLS and Manual. The images depict interactive TLS (3D point cloud)-based height measurement within the CloudCompare software.


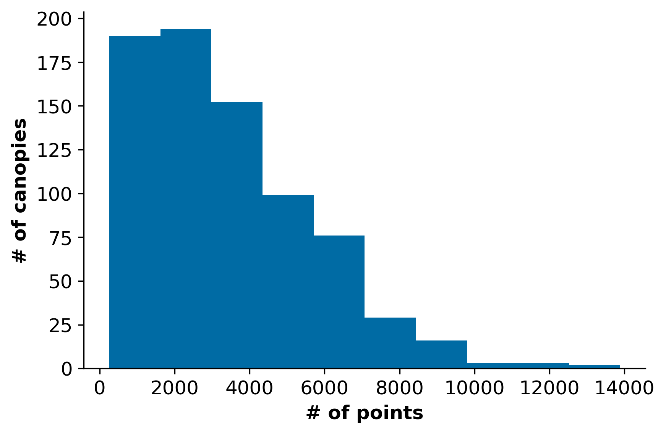


**S4.** This histogram displays the distribution of canopies based on the number of points they contain. The data pertains to 464 soybean cultivars including 450 plant introduction (PI) lines that were studied, representing a diverse range of maturities, seed weights, and stem terminations, and originating from 35 different countries. Data was obtained from one or two replicates per cultivar.
